# Minimally interactive segmentation of soft-tissue tumors on CT and MRI using deep learning

**DOI:** 10.1007/s00330-024-11167-8

**Published:** 2024-11-19

**Authors:** Douwe J. Spaanderman, Martijn P. A. Starmans, Gonnie C. M. van Erp, David F. Hanff, Judith H. Sluijter, Anne-Rose W. Schut, Geert J. L. H. van Leenders, Cornelis Verhoef, Dirk J. Grünhagen, Wiro J. Niessen, Jacob J. Visser, Stefan Klein

**Affiliations:** 1 Department of Radiology and Nuclear Medicine, Erasmus MC, Rotterdam, The Netherlands; 2 Department of Surgical Oncology, Erasmus MC Cancer Institute, Rotterdam, The Netherlands; 3 Department of Medical Oncology, Erasmus MC Cancer Institute, Rotterdam, The Netherlands; 4 Department of Pathology, Erasmus MC Cancer Institute, Rotterdam, The Netherlands; 5 Faculty of Medical Sciences, University of Groningen, Groningen, The Netherlands

**Keywords:** Soft-tissue neoplasms, Diagnostic imaging, Deep learning, Magnetic resonance imaging, Tomography (X-ray computed)

## Abstract

**Background:**

Segmentations are crucial in medical imaging for morphological, volumetric, and radiomics biomarkers. Manual segmentation is accurate but not feasible in clinical workflow, while automatic segmentation generally performs sub-par.

**Purpose:**

To develop a minimally interactive deep learning-based segmentation method for soft-tissue tumors (STTs) on CT and MRI.

**Material and methods:**

The interactive method requires the user to click six points near the tumor’s extreme boundaries in the image. These six points are transformed into a distance map and serve, with the image, as input for a convolutional neural network. A multi-center public dataset with 514 patients and nine STT phenotypes in seven anatomical locations, with CT or T1-weighted MRI, was used for training and internal validation. For external validation, another public dataset was employed, which included five unseen STT phenotypes in extremities on CT, T1-weighted MRI, and T2-weighted fat-saturated (FS) MRI.

**Results:**

Internal validation resulted in a dice similarity coefficient (DSC) of 0.85 ± 0.11 (mean ± standard deviation) for CT and 0.84 ± 0.12 for T1-weighted MRI. External validation resulted in DSCs of 0.81 ± 0.08 for CT, 0.84 ± 0.09 for T1-weighted MRI, and 0.88 ± 0.08 for T2-weighted FS MRI. Volumetric measurements showed consistent replication with low error internally (volume: 1 ± 28 mm^3^, *r* = 0.99; diameter: − 6 ± 14 mm, *r* = 0.90) and externally (volume: − 7 ± 23 mm^3^, *r* = 0.96; diameter: − 3 ± 6 mm, *r* = 0.99). Interactive segmentation time was considerably shorter (CT: 364 s, T1-weighted MRI: 258s) than manual segmentation (CT: 1639s, T1-weighted MRI: 1895s).

**Conclusion:**

The minimally interactive segmentation method effectively segments STT phenotypes on CT and MRI, with robust generalization to unseen phenotypes and imaging modalities.

**Key Points:**

***Question*** *Can this deep learning-based method segment soft-tissue tumors faster than can be done manually and more accurately than other automatic methods?*

***Findings*** *The minimally interactive segmentation method achieved accurate segmentation results in internal and external validation, and generalized well across soft-tissue tumor phenotypes and imaging modalities.*

***Clinical relevance*** *This minimally interactive deep learning-based segmentation method could reduce the burden of manual segmentation, facilitate the integration of imaging-based biomarkers (e.g., radiomics) into clinical practice, and provide a fast, semi-automatic solution for volume and diameter measurements (e.g., RECIST).*

## Introduction

Soft-tissue tumors (STTs) are rare tumors with a broad range of differentiation that can occur in a large variety of locations in the body. STT progression is highly variable across patients [1, 2]. The 3D delineation of STTs, i.e., segmentation, is needed for various purposes, such as targeted (neo)adjuvant radiotherapy planning [3], computation of quantitative imaging biomarkers (radiomics) [4–7], and calculation of Response Evaluation Criteria in Solid Tumors (RECIST) [8]. Currently, these segmentations would have to be made manually, which is a substantial burden on the physician’s time, drives healthcare costs, and is observer-dependent. Therefore, there is a need for more time-efficient, automated segmentation methods in clinical practice.

Fully automatic segmentation methods using deep learning have been shown to be successful in various applications in medical imaging [9]. However, their adoption in STTs has been limited due to the vast range of STT phenotypes, locations, and imaging modalities [10], which makes it difficult to train a fully automatic segmentation method that generalizes across all STT patients [10, 11]. As a result, the performance of automatic segmentation methods will remain sub-par. A potential solution could be to allow a minimal amount of manual interaction, leveraging the radiologist’s knowledge to guide the segmentation and thereby improve generalizability, while maintaining practical efficiency in a clinical setting [12].

The aim of this work was to develop and evaluate a minimally interactive deep-learning method for STT segmentation on CT and MRI. To this end, a previously proposed framework [12] was adopted, the methodology was optimized using best practices from the state-of-the-art nnU-Net framework [9], and it was trained on a heterogeneous public dataset. For comparison, the previously proposed interactive segmentation method without any modifications [12] and a fully automatic segmentation method using the nnU-Net framework [9] were also implemented. Methods were compared to manual reference segmentations on another independent public dataset. Finally, volume and diameter measurements were conducted to analyze the use of minimally interactive segmentation for clinical measurements.

## Materials and methods

### Study sample

In this study, the only two publicly available datasets including STTs were used. The characteristics of the datasets are described in Table 1, and properties of image acquisition protocols are summarized in Table S1.

**Table 1:** Descriptive characteristics of the datasets used in this study

Characteristics	WORC training dataset (*n* = 412) [13]	WORC test dataset (*n* = 102) [13]	TCIA test dataset (*n* = 51) [14]
Age (years)			
Range	5–93	1–86	16–83
Mean ± SD	57 ± 17	55 ± 20	55 ± 17
Sex			
Female	206 (50%)	54 (53%)	27 (53%)
Male	206 (50%)	48 (47%)	24 (47%)
Location			
Lower extremities	156 (38%)	34 (33%)	47 (92%)
Upper extremities	27 (7%)	7 (7%)	4 (8%)
Head and neck	14 (3%)	4 (4%)	-
Intra-abdominal	159 (9%)	39 (38%)	-
Retroperitoneum and pelvis	3 (1%)	-	-
Trunk	53 (13%)	18 (18%)	-
Volume (mm^3^)			
Range	0.6–7944	1.1–3138	17–2361
Mean ± SD	379 ± 837	305 ± 505	474 ± 499
Modality			
CT	158 (38%)	39 (38%)	51 (100%)
T1-weighted MRI	254 (62%)	63 (62%)	51 (100%)
T2-weighted fat-suppressed MRI	-	-	51 (100%)
Phenotype			
Lipoma	46 (11%)	11 (11%)	-
Well-differentiated liposarcoma	46 (11%)	11 (11%)	-
Desmoid-type fibromatosis	58 (14%)	14 (14%)	-
Myxofibrosarcoma	49 (12%)	12 (12%)	-
Myxoid liposarcoma	29 (7%)	8 (8%)	-
Gastrointestinal stromal tumor	100 (24%)	24 (24%)	-
Schwannoma	18 (4%)	5 (5%)	-
Leiomyosarcoma	46 (11%)	12 (12%)	10 (20%)
Leiomyoma	20 (5%)	5 (5%)	-
Liposarcoma	-	-	11 (21%)
Fibrosarcoma	-	-	1 (2%)
Synovial sarcoma	-	-	5 (10%)
Malignant fibrous histiocytoma	-	-	17 (33%)
Extraskeletal bone sarcoma	-	-	4 (8%)
Other	-	-	3 (6%)

Except where indicated, data are the number of patients (percentages)

*SD* standard deviation

**Fig. 1:**
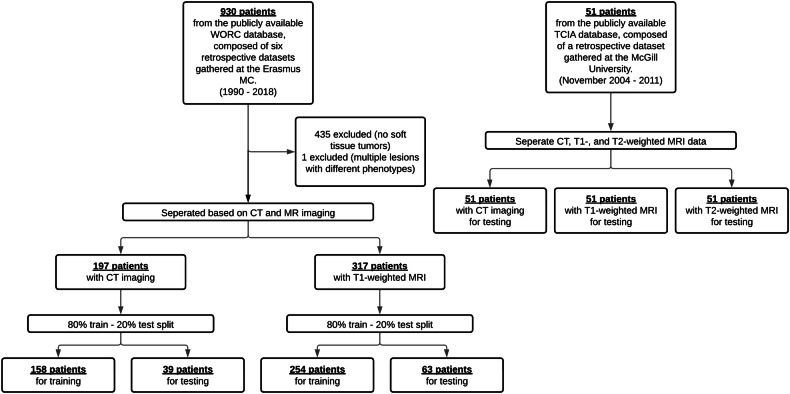
Flowchart showing the two publicly available, retrospective, datasets used in this study and the exclusion criteria

For model development and internal validation, all STTs from the public, retrospective, multi-center WORC database were used, which includes 514 patients with either CT or T1-weighted MRI (Fig. 1) [13]. The WORC database includes reference tumor delineations obtained through manual segmentation on the T1-weighted MRI or CT scans by various clinicians under the supervision of musculoskeletal radiologists (4-5 years of experience). For both CT and MRI, the dataset was split stratified on STT phenotype, using 80 percent for model development and 20 percent for internal validation, denoted as the WORC training and test datasets, respectively.

For external validation, the publicly available STT data of 55 patients released on The Cancer Imaging Archive (TCIA) was used, referred to as the TCIA test dataset (Fig. 1) [14]. For all patients, CT, T1-weighted, and T2-weighted fat-saturated (FS) MRI are available. Reference segmentations were manually made by one expert radiation radiologist on the T2-weighted FS MRI scans. Additionally, registered segmentation to CT and T1-weighted MRI were also included. The TCIA test dataset includes one modality (T2-weighted FS MRI) and five tumor phenotypes (Table 1) that are not available in the WORC datasets, enabling the assessment of generalizability in unseen conditions.

### Interactive segmentation

**Fig. 2:**
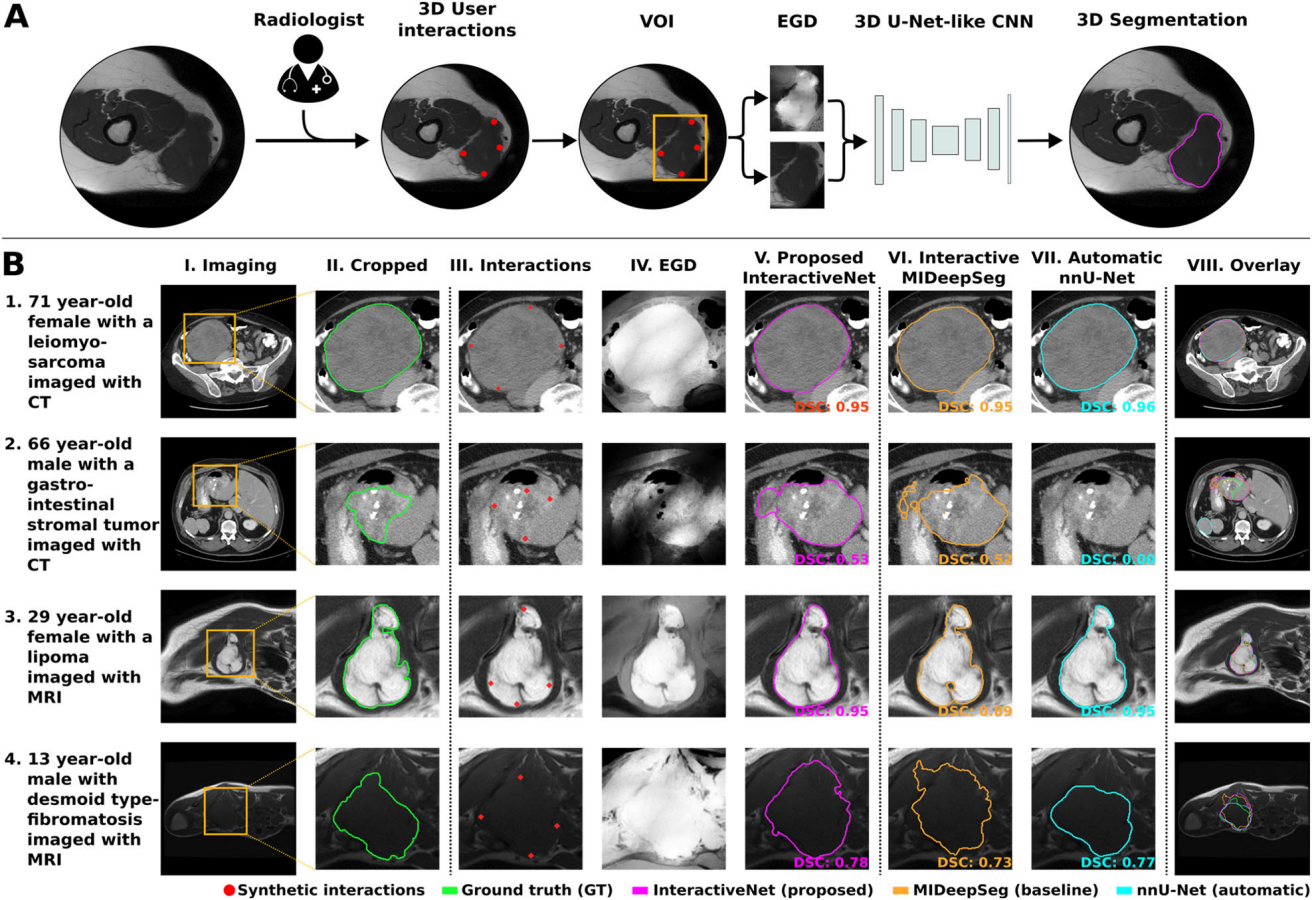
**A** Schematic overview of the interactive framework, based on [12], used in this study. For visualization purposes, one 2D slice in the transverse plane is shown, while all data are 3D images, and all operations are performed in 3D. In the interactive segmentation pipeline, a clinician has to draw six interior margin points in the 3D object. Next, using these interactions, the volume of interest (VOI) is extracted, and the exponentialized geodesic distance map (EGD) is calculated. The cropped image and the EGD map are concatenated and fed through a 3D U-Net-like convolutional neural network (CNN). **B** Qualitative comparison of interactive and fully automatic segmentation methods in four different patients with STT in the WORC test dataset; The images show (I) CT imaging (rows 1 and 2) or T1-weighted MRI (rows 3 and 4) in the transverse plane, (II) zoomed-in image with reference segmentation for visualization purposes, (III) interior margin points derived synthetically, (IV) EGD map derived from the interior margin points, (V) predicted segmentation from proposed InteractiveNet, VI) predicted segmentation from interactive MIDeepSeg, (VII) predicted segmentation from fully automatic nnU-Net, (VIII) segmentation comparison in full image

The interactive segmentation method (InteractiveNet) is based on the framework by Luo et al [12], as it has been designed for the medical domain, requires limited user interactions, and has been shown to generalize well to unseen objects. In the method (Fig. 2A), users click six points near the extreme boundaries of the 3D object of interest, i.e., two extreme interior margin points in three planes (transversal, coronal, sagittal). Using these interior margin points, the image is cropped to a volume of interest (VOI) to aid the model in tumor localization. A minimum of six points inside the object are required to ensure that the VOI encapsulates the entire 3D object. The cropping boundaries are slightly relaxed in order to encapsulate the whole object. The interior margin points are also used to calculate an exponentialized geodesic distance (EGD) map, which provides a similarity map based on the intensity and spatial differences between surrounding voxels and user clicks. We hypothesize that the EGD will “highlight” the tumor voxels based on the intensity differences with the surrounding tissue. Together, the cropped image and EGD map are used as input for a 3D convolutional neural network (CNN).

In order to provide more accurate segmentation results, the original work from Luo et al [12], was combined with best practices from the state-of-the-art self-configuring nnU-Net framework [9], including preprocessing, network architecture, training, post-processing, and ensembling. Implementation details can be found in Supplementary Materials A. To evaluate the benefits of these modifications, the newly developed InteractiveNet was compared to the method described in the original work by Luo et al 2021, referred to as “MIDeepSeg” [12]. Since the original code for model training was not available, MIDeepSeg was reimplemented using the implementation details provided [12].

### Fully automatic segmentation

To compare the performance of InteractiveNet, a fully automatic segmentation method using nnU-Net was trained [9]. nnU-Net deploys a 3D CNN on the whole volume to locate and segment the tumor. Prior to this work, nnU-Net has not yet been applied to STTs.

### Experimental setup

Separate segmentation models were trained for CT and T1-weighted MRI, both for the interactive and fully automatic approaches. First, the models were validated on the WORC test dataset. Second, external validation, including assessment of generalization to unseen phenotypes and T2-weighted FS MRI sequences not encountered during training, was performed on the TCIA test dataset. Here, the models trained on T1-weighted MRIs were used to generate segmentations for T2-weighted FS MRI.

For both the WORC and TCIA datasets, the six interior margin points per image required for the interactive segmentation method were synthetically generated based on the reference segmentation. First, extreme points were identified along all axes using reference segmentation. Next, to make sure the points were inside the object, these points were moved five voxels inwards in-plane, and in case of anisotropic images (maximum axis spacing/minimum axis spacing > 3), in the out-of-plane dimension by 1 voxel.

In order to further validate synthetic interactions, a musculoskeletal radiologist (8 years of experience) and a medical student also performed real user interactions on the WORC test dataset. A medical student was chosen to assess the need for expert radiological knowledge to perform these interactions. They were provided only the images and blinded to clinical data, including STT phenotype. The users had not seen these images prior to annotation, and the reference segmentation was not shown during annotation. We evaluated: (1) The model outputs generated from these real user interactions compared to the reference segmentation; (2) user-provided quality scores based on four scales (Excellent, Sufficient, Insufficient, Incorrect, see Table S2 for details); and (3) time annotate (including lesion identification and the placement of six points), run the method, and score segmentation quality.

### Statistical analysis

Segmentation performance was evaluated using the sensitivity, intersection over union (IOU), and DSC [15]. To determine differences in DSC between InteractiveNet and both nnU-Net and MIDeepSeg, the Wilcoxon signed-rank test was used. *p*-values < 0.05 were considered statistically significant.

Pyradiomics was used to calculate the volume and maximum diameter in the transverse plane of the predicted and reference segmentation [16]. Agreement between these measurements across segmentations was assessed using Bland-Altman plots and the Pearson correlation coefficient (*r*) [17].

## Results

### Comparison of WORC test dataset of interactive, fully automatic, and reference segmentations

**Fig. 3:**
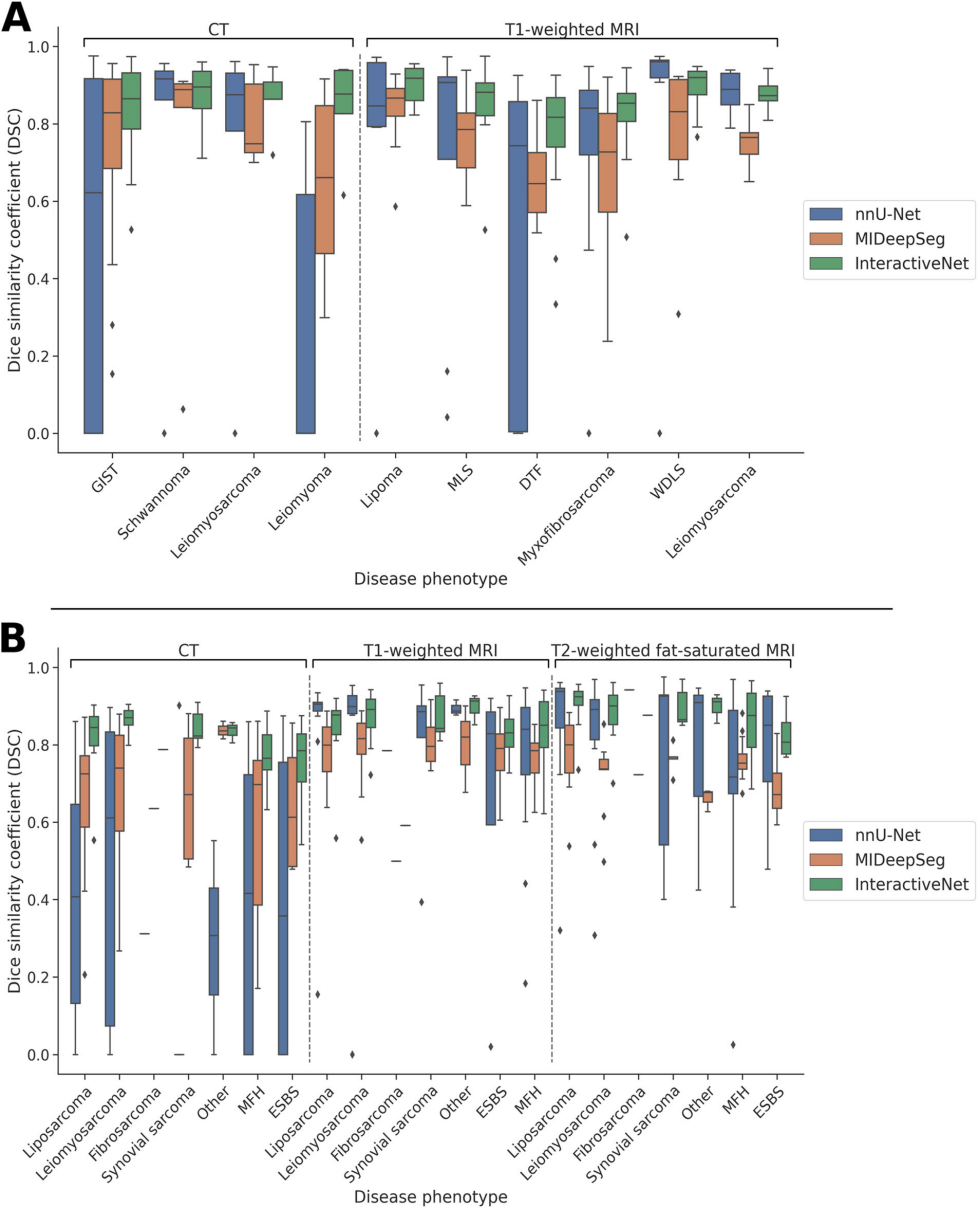
Quantitative results from automatic and interactive segmentation of STTs on the (**A**) WORC test dataset, and (**B**) TCIA test dataset. Box-and-whisker plot, visualizing the median, quartiles, and potential outliers, for the dice similarity coefficient (DSC) results of fully automatic nnU-Net (blue), interactive “MIDeepSeg” (orange), and interactive proposed “InteractiveNet” (green) segmentation methods for different phenotypes on CT and MRI. Higher DSC means better segmentation accuracy. MLS, myxoid-liposarcoma; DTF, desmoid-type fibromatosis; WDLS, well-differentiated liposarcoma; GIST, gastrointestinal stromal tumor; MFH, malignant fibrous histiocytoma; and ESBS, extraskeletal bone sarcoma

Segmentation from InteractiveNet resulted in a higher mean DSC and lower SD (CT: 0.85 ± 0.11, T1-weighted MRI: 0.84 ± 0.12) than from nnU-Net (CT: 0.52 ± 0.43, T1-weighted MRI: 0.71 ± 0.35). Differences were statistically significant for CT (*p* < 0.001), whereas no significant differences were observed for T1-weighted MRI (*p* = 0.15). InteractiveNet demonstrated statistically significant improvements over MIDeepSeg, which scored 0.74 ± 0.23 for CT (*p* < 0.001) and 0.74 ± 0.15 for T1-weighted MRI (*p* < 0.001) (Fig. 3A and Table 2; Table S3).

**Table 2:** Phenotype-specific agreement between nnU-Net, MIDeepSeg, and InteractiveNet with reference segmentation

	CT			T1-weighted MRI			T2-weighted FS MRI		
	nnU-Net	MIDeepSeg	InteractiveNet	nnU-Net	MIDeepSeg	InteractiveNet	nnU-Net	MIDeepSeg	InteractiveNet
WORC test dataset									
GIST	0.49 ± 0.43*	0.75 ± 0.22*	0.84 ± 0.12						
Schwannoma	0.73 ± 0.37	0.72 ± 0.37	0.87 ± 0.09						
Leiomyoma	0.28 ± 0.35	0.64 ± 0.26	0.84 ± 0.01						
Leiomyosarcoma	0.71 ± 0.36	0.81 ± 0.11	0.86 ± 0.08	0.88 ± 0.05	0.75 ± 0.06*	0.88 ± 0.04			
Lipoma				0.73 ± 0.35	0.84 ± 0.10*	0.90 ± 0.05			
MLS				0.72 ± 0.36	0.77 ± 0.12	0.84 ± 0.13			
DTF				0.49 ± 0.39*	0.66 ± 0.10*	0.75 ± 0.17			
Myxofibrosarcoma				0.69 ± 0.33	0.67 ± 0.20*	0.82 ± 0.11			
WDLS				0.86 ± 0.27	0.77 ± 0.18*	0.89 ± 0.06			
*Total*	0.52 ± 0.43*	0.74 ± 0.23*	0.85 ± 0.11	0.71 ± 0.35	0.74 ± 0.15*	0.84 ± 0.12			
TCIA test dataset									
Liposarcoma	0.40 ± 0.30*	0.66 ± 0.19*	0.82 ± 0.09	0.83 ± 0.22	0.79 ± 0.07*	0.84 ± 0.10	0.84 ± 0.18	0.78 ± 0.10*	0.90 ± 0.06
Leiomyosarcoma	0.49 ± 0.36*	0.66 ± 0.21*	0.87 ± 0.03	0.82 ± 0.27	0.79 ± 0.10*	0.87 ± 0.07	0.81 ± 0.20	0.72 ± 0.09*	0.88 ± 0.07
Fibrosarcoma^a^	0.31	0.64	0.79	0.78	0.50	0.59	0.94	0.72	0.88
Synovial sarcoma	0.18 ± 0.36	0.67 ± 0.16	0.84 ± 0.04	0.79 ± 0.20	0.81 ± 0.07	0.87 ± 0.06	0.75 ± 0.24	0.76 ± 0.03	0.90 ± 0.05
MFH	0.39 ± 0.33*	0.57 ± 0.23*	0.77 ± 0.07	0.77 ± 0.20	0.76 ± 0.06*	0.83 ± 0.10	0.72 ± 0.23	0.76 ± 0.05*	0.86 ± 0.09
ESBS	0.40 ± 0.40	0.64 ± 0.16	0.75 ± 0.13	0.65 ± 0.37	0.77 ± 0.11*	0.83 ± 0.07	0.78 ± 0.18	0.69 ± 0.09	0.83 ± 0.06
Other	0.29 ± 0.23	0.84 ± 0.02	0.84 ± 0.02	0.89 ± 0.02	0.80 ± 0.09	0.90 ± 0.03	0.76 ± 0.24	0.66 ± 0.02	0.90 ± 0.03
*Total*	0.38 ± 0.34*	0.64 ± 0.21*	0.81 ± 0.08	0.79 ± 0.23	0.78 ± 0.09*	0.84 ± 0.09	0.78 ± 0.22*	0.75 ± 0.08*	0.88 ± 0.08

Data are mean ± standard deviation (SD) for the dice similarity coefficient (DSC)

*FS* fat-saturated, *MLS* myxoid-liposarcoma, *DTF* desmoid-type fibromatosis, *WDLS* well-differentiated liposarcoma, *GIST* gastrointestinal stromal tumor, *MFH* malignant fibrous histiocytoma, *ESBS* extraskeletal bone sarcoma

* *p* < 0.05 (Wilcoxon signed-rank test of DSC compared to InteractiveNet)

^a^ Standard deviation could not be calculated as only one fibrosarcoma was present in the TCIA test dataset

Examples of interactive and fully automatic segmentations, including the synthetic interactions and EGD map, are shown in Fig. 2B. Qualitative analysis showed that poor contrast between the tumor and surrounding tissue sometimes led to low DSC using either method (Fig. 2B). Additionally, all methods had difficulties with tumor boundaries when tumors were irregular and lobulated. nnU-Net had difficulties with large fields of view, especially in CT, as tumor detection showed to be difficult, sometimes resulting in no segmentation (CT: *n* = 5/39, T1-weighted MRI: *n* = 5/62), or segmentation of a different object (CT: *n* = 10/39, T1-weighted MRI: *n* = 3/62). Even after removing the 23 outliers on which nnU-Net failed to segment the correct lesion, Interactivenet outperformed the fully automatic method in terms of DSC, with a higher overall median, smaller interquartile range, and fewer outliers (Fig. S1).

Supplementary Materials B provides detailed information on the interobserver variability in manual segmentations by two human annotators, the effect of user variability simulated by introducing random noise into synthetic interactions, and a comparison with real user interactions.

### External validation on TCIA test dataset including unseen tumor phenotypes and unseen imaging sequences

InteractiveNet was able to segment unseen phenotypes on both CT and T1-weighted MRI with similar DSC to the phenotypes in the WORC test dataset (CT: 0.81 ± 0.09, T1-weighted MRI: 0.84 ± 0.09) (Fig. 3B and Table 2; Table S3). nnU-Net performed similarly for unseen phenotypes on T1-weighted MRI (DSC: 0.81 ± 0.23); however, it failed more often to segment tumors on CT (DSC: 0.38 ± 0.34) compared to interactive segmentation (T1-weighted MRI: *p* = 0.54, CT: *p* < 0.001). InteractiveNet also provided significantly improved results over MIDeepSeg for CT (DSC: 0.64 ± 0.21, *p* < 0.001) and T1-weighted MRI (DSC: 0.78 ± 0.09, *p* < 0.001).

The detection of unseen phenotypes on the unseen modality, T2-weighted FS MRI, improved slightly for InteractiveNet compared to T1-weighted MRI (DSC: 0.88 ± 0.08). nnU-Net provided slightly worse results on T2-weighted FS MRI (DSC: 0.78 ± 0.22) compared to InteractiveNet (*p* = 0.02). Similarly, InteractiveNet provided improved results compared to MIDeepSeg (DSC: 0.75 ± 0.08, *p* < 0.001). Better contrast between tumor and surrounding tissue on T2-weighted FS in comparison to T1-weighted MRI may explain the slightly better segmentation results on T2-weighted FS MRI such as shown in example (Fig. S2).

### Agreement of volume and diameter measurements

**Fig. 4:**
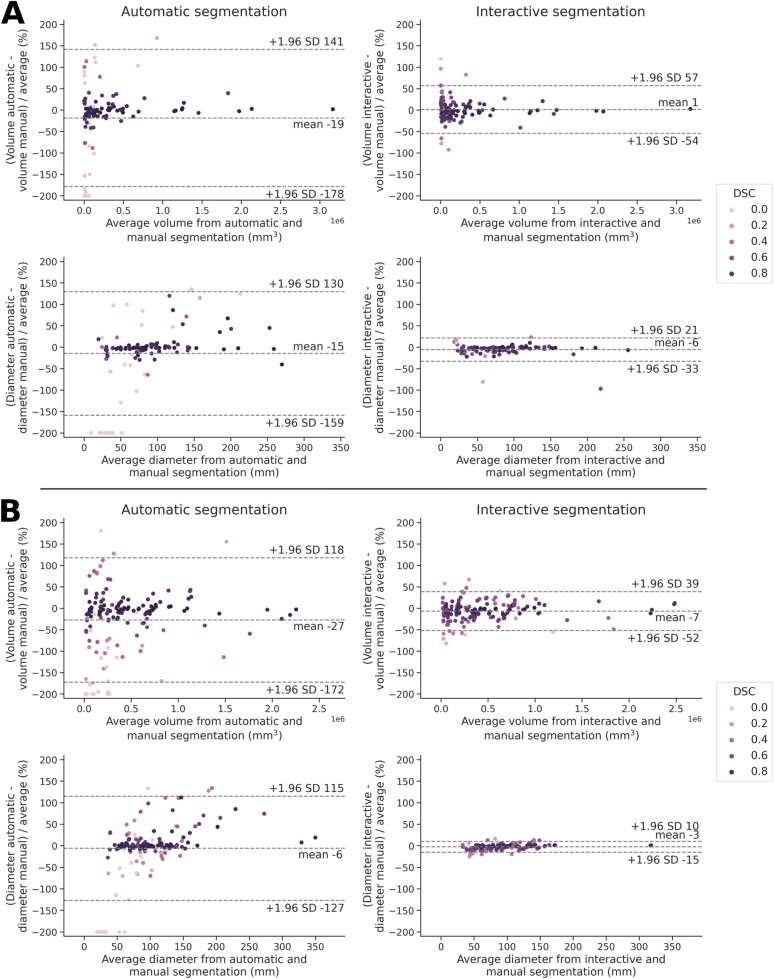
Bland-Altman plots for percentage volume and diameter measurements from automatic and interactive compared to reference segmentations of STTs on (**A**) the WORC test dataset, (**B**) the TCIA test dataset. Every dot represents a patient. Patients are color-coded based on their achieved dice similarity coefficient (DSC) using the respective method, i.e., automatic or interactive segmentation

Volume and diameter measurements based on InteractiveNet showed low errors compared to the reference segmentation, both in the WORC test dataset (mean ± SD volume error: 1 ± 28 mm^3^, *r* = 0.99; diameter: −6 ± 14 mm, *r* = 0.90) and in the TCIA test dataset (volume: −7 ± 23 mm^3^, *r* = 0.96; diameter: −3 ± 6 mm, *r* = 0.99) (Fig. 4 and S3).

### Time-efficiency of segmentation methods

Respectively, the radiologist and medical student required on average 258 seconds (s) and 364 s for CT, and 122 s and 388 s for T1-weighted MRI to perform interactive segmentation on the WORC dataset, which was considerably shorter than manual segmentation (CT: 1639 s, T1-weighted MRI: 1895s) (Table 3) [5–7].

**Table 3:** Time to perform minimally interactive segmentation, manual segmentation, and fully automatic segmentation

	CT				T1-weighted MRI			
	Radiologist	Medical student	Manual^a^	Fully automatic^b^	Radiologist	Medical student	Manual^a^	Fully automatic^b^
Annotation^c^	146 ± 103	247 ± 608			87 ± 99	210 ± 538		
Preprocessing	21 ± 12	21 ± 12			2.8 ± 2.2	2.7 ± 2.4		
Model inference	1.3 ± 1.8	1.3 ± 1.8			0.4 ± 0.5	0.4 ± 0.4		
Post-processing	21 ± 10	17 ± 7.6			2.0 ± 1.6	2.0 ± 1.7		
Evaluation	70 ± 84	42 ± 37			27 ± 23	160 ± 774		
User total	216 ± 135	325 ± 617			117 ± 106	383 ± 840		
Method total	43 ± 19	39 ± 17		1940 ± 1113	5.2 ± 3.7	5.1 ± 4.0		100 ± 95
Total	258 ± 135	364 ± 626	1895 ± 1804		122 ± 107	388 ± 840	1639 ± 1397	

Data are mean ± standard deviation (SD) in seconds. Results are reported on the WORC test dataset. User total: annotation + evaluation; method total: preprocessing + model inference + postprocessing

^a^ Time to create the manual reference segmentation is also reported for comparison

^b^ Total inference time of the fully automatic segmentation method is also reported for comparison

^c^ Annotation time included lesion identification and the placement of six points

## Discussion

In this study, a deep-learning method for minimally interactive segmentation of STTs on CT and MRI was developed. Upon internal validation in the WORC test dataset, InteractiveNet achieved a high degree of overlap with the reference segmentation of clinicians for nine STT phenotypes (CT: 0.85 ± 0.109, T1-weighted MRI: 0.84 ± 0.121), with higher mean DSC and lower SD compared to fully automatic segmentation (nnU-Net) and MIDeepSeg. In the external TCIA test dataset, InteractiveNet showed good generalizability to unseen phenotypes and unseen MRI sequences (CT: 0.81 ± 0.092, T1-weighted MRI: 0.84 ± 0.092, T2-weighted FS MRI: 0.88 ± 0.075). In addition, there was low error compared to the reference segmentation for volume and diameter calculations between interactive and reference segmentations.

In Supplementary Materials C, the high potential for time-efficient segmentation in STTs (e.g., morphological measurements, radiomics biomarkers) and additional considerations regarding the clinical implementation of the minimally interactive segmentation method (e.g., combination with automatic segmentation) are detailed.

Two previous studies have focused on deep learning-based segmentation for STT. First, a study using the TCIA database showed accurate results (DSC: 0.88) when combining FDG-PET, CT, and T2-weighted FS MRI for automatic segmentation [18]. However, this study only assessed STT in the extremities, lacked external validation, and required all three modalities to provide accurate segmentations. Next, recent work achieved accurate automatic segmentation of lipomatous tumors (DSC: 0.80 ± 0.184) but lacked external validation and focused on a single STT type [19]. Here, an interactive segmentation method with improved segmentation results that generalize well to different STT phenotypes is provided.

This study has two main limitations. First, the method was evaluated on 14 STT phenotypes, while there are over 100 histological phenotypes. Although this study shows that the method generalized well to five unseen STT phenotypes, this is no guarantee that it also translates to other STT phenotypes or even other cancer types beyond STT; hence, future work should investigate the further generalization to other tumor types. Second, either CT, T1-weighted, or T2-weighted FS MRI was used as model input; a multimodal approach using different sequences simultaneously may improve results further [18].

In conclusion, the minimally interactive deep learning-based segmentation method can accurately generate segmentations for a wide variety of STTs in different body parts imaged with CT or MRI [20]. Therefore, this method could reduce the burden of manual segmentation for targeted (neo)adjuvant therapy, enable the integration of imaging-based biomarkers (e.g., radiomics) into clinical practice, and provide a fast, semi-automatic solution for volume and diameter measurements in the clinic. Future research also includes an extension of the method to other cancer types beyond STT. Due to the wide variety of STT phenotypes, locations, and imaging appearance, we expect that the method may even work directly on other (similar) tumors without any adjustments.

## Supplementary information

ELECTRONIC SUPPLEMENTARY MATERIAL

Supplementary Movie 1

## Data Availability

All data used in this study is publicly available [13, 14].

## Abbreviations

CNN: Convolutional neural network
DSC: Dice similarity coefficient
EGD: Exponentialized geodesic distance
FS: Fat-saturated
RECIST: Response Evaluation Criteria in Solid Tumors
STT: Soft-tissue tumor
TCIA: The Cancer Imaging Archive
VOI: Volume of interest

## Code availability

To facilitate the use of InteractiveNet, a graphical user interface (GUI), a video tutorial (Video S1), and the code for model training and evaluation are provided [18]. This repository included the code to replicate the experiments conducted in this study. Model weight required for soft-tissue tumor segmentation prediction are also provided.
