# Supplementary material for: Minimally interactive segmentation of soft-tissue tumors on CT and MRI using deep learning
Source: Eur Radiol. 2024 Nov 19;35(5):2736–45. doi: 10.1007/s00330-024-11167-8 (PMC12021718; doi:10.1007/s00330-024-11167-8)
Supplement: Supplementary file 1 — ELECTRONIC SUPPLEMENTARY MATERIAL [file 330_2024_11167_MOESM1_ESM.docx]

**Supplementary Material A: Details on InteractiveNet**

For the preprocessing, network architecture, training, and post-processing, most of the best practices found in the nnU-Net method were followed (9). The design choices of the interactive segmentation implementation are divided into 1) fixed parameters, which are predefined and do not change based on the application; 2) rule-based parameters, which are based on the characteristics of the dataset; 3) empirical parameters, which are determined based on achieved results.

The code and model weights for training and evaluation the self-configuring interactive segmentation method are provided (18), including a graphical user interface, implemented in MONAI Label, in which the clinician can draw the six interior margin points and run the interactive segmentation method (21). The code to train MIDeepSeg is also provided (18).

The segmentation methods were implemented in Python (3.9.5) and Pytorch (1.21.1) using a NVIDIA A40 (48GB) GPU.

**Fixed parameters**

*Volume of interest selection*. The same strategy as Luo et al, 2021 (12), where volume of interest (VOI) extraction is based on the interior margin points from the user, was used. In order to make sure that the complete object is incorporated in the image and the dynamics at the boundary of the object is fully captured, the VOI is expanded slightly by a relaxation factor of 0.1 times the size of the VOI in each axis. Finally, if the input is not divisible by 2 to the power of the number of downsampling operations, the VOI is further extended, possibly using zero-padding in the case that the VOI falls outside the image.

*Deep learning model architecture*. A convolutional neural network (CNN), more specifically a 3D U-Net-like network with instance normalization, leaky ReLU, and deep supervision similar to nnU-Net (9), was used. Down- and upsampling are implemented as respectively a stride and transpose convolution. In line with the original work from Luo et al., 2021 (12), as the images are cropped to the VOI and are therefore already much smaller than the original image, the number of feature maps were reduced to start at 4 and double/halve with each down-/upsampling operation, in order to limit the required memory consumption, and achieve real-time inference.

*Training*. A five-fold cross-validation was used to train five CNNs. These models are trained for 1000 epochs, using a poly learning rate scheduler (initial learning rate = 0.01, learning rate decay = (1−epoch/epoch_max_)^0.9^), Dice + cross-entropy loss, stochastic gradient descent with Nesterov momentum (μ = 0.99), and deep supervision with additional auxiliary losses in the decoder for all but the lowest two resolution. During training, inputs were randomly augmented using rotations, zooms, and flips. Additionally, the image is randomly altered using Gaussian noise, Gaussian smoothing, intensity scaling, and contrast adjustment. These configurations are all in line with nnU-Net. However, due to dealing with unequal-sized images, batch sizes larger than 1 cannot be created. To stabilize training, gradient accumulation was used (n = 4), i.e. updating the weights after four iterations.

*Inference*. all five models are used during inference time, and combine the results with mean ensembling. Also, for each model test time augmentation was used, where the image is flipped in all directions, e.g. eight different flips for 3D images, and combine the results with mean ensembling.

**Rule-based parameters**

The rule-based parameters are based on the characteristics of the training dataset. For prediction on new samples, the same rule-based parameters are used as during training.

*Resampling*. The physical space a voxel represents (spacing) is often heterogeneous between different medical images, which needs to be homogenized before providing the image to the CNN. Here the nnU-Net resampling method is used, in which the median spacing for each axis in the training dataset is used as target spacing. In the case of anisotropic (maximum axis spacing / minimum axis spacing > 3) resampling, which is the case for the WORC dataset, the tenth percentile is taken for the lowest resolution axis. This is done to conserve as much resolution in this axis as possible. Images are resampled with third-order spline and anisotropic out-of-plane dimension with nearest neighbor. Similarly, segmentations are resampled with linear interpolation and out-of-plane with nearest neighbor. The interactions from the clinician also have to be resampled to the new voxel spacing. Therefore, first the interactions are changed to real-world coordinates and subsequently these coordinates are mapped to the location in the resized image.

*Normalization*. The same normalization is used as deployed by nnU-Net, in which for CT, normalization is done by percentile clipping and z-scoring based on the voxels in the tumor (foreground voxels) in the training dataset. For MRI, normalization is done per image using z-scoring. In contrast to nnU-Net, normalization for both modalities is done based on the extracted VOI only.

*Network topology*. In line with nnU-Net, by default a 3 x 3 x 3 kernel size for convolution is used. However, when dealing with anisotropic images, pseudo-3D kernels, e.g. 3 x 3 x 1, can be deployed to deal with resolution discrepancy. Similar to nnU-Net, resolution discrepancy is defined by a spacing ratio larger than two. Given the anistotropic images in the WORC dataset, the initial two convolution operations are done using this 3 x 3 x 1 convolution.

**Empirical parameters**

*Post-processing*. Post-processing involves the utilization of either the largest connected component, filling small holes, or both. The effect of post-processing on improving the DSC of the segmentation compared to the reference segmentation is assessed. This is evaluated by comparing the DSC in cross-validation splits both with and without post-processing methods. For CT and MRI data in the WORC dataset, both these post-processing steps turned out to be effective, and were thus employed.

**Supplementary Material B: Results**

**Interobserver variability between manual annotators**

For a subset of the WORC dataset (n=60), the interobserver DSC was reported between two manual annotators (CT: 0.84±0.20, T1-weighted MRI: 0.77±0.20) (5-7). This shows that the performance of the interactive segmentation matched or exceeded intra-observer variability (CT: 0.85±0.11, T1-weighted MRI: 0.84±0.12). Moreover, the standard deviation of the interactive segmentation was lower against the reference segmentation, compared to the two manual annotators, suggesting that interactive segmentation reduces variability among produced segmentations.

**Mimicking user variability with random noise on synthetic interactions**

To determine the impact of noise, for every sample in the WORC test dataset, 50 additional noisy synthetic interactions were created. These interactions were generated by introducing random noise, defined as displacements within the range of [-3 to 3] voxels, along one or all axes, while ensuring that the generated interactions remained within the bounds of the image. The mean and standard deviation in DSC change (ΔDSC) between noisy interactions and original interactions was calculated. For both CT (ΔDSC: -0.023 ± 0.080) and MRI (ΔDSC: -0.028 ± 0.078), marginal difference in DSC was observerd with the noisy synthetic interactions, suggesting that variations in interactions have minimal impact on segmentation outcomes.

**Comparison on WORC test dataset of synthetic and real user interactions**

InteractiveNet was compared with synthetic and real user interactions on the WORC test dataset **(Figure S4, Table S4)**. The musculoskeletal radiologist’s interactions performed significantly worse on the T1-weighted MRI data (DSC: 0.75 ± 0.25) in comparison to the synthetic interactions (DSC: 0.84 ± 0.12, p=0.07), whereas the medical student scored significantly worse on the CT data (DSC: 0.60 ± 0.40) in comparison to the synthetic interactions (DSC: 0.85 ± 0.11, p=0.02). The difference in DSC between the two users and the synthetic interactions was mostly explained by the number of wrongly identified objects (radiologist: n=7/102, student: n=16/102). Excluding these samples increased the DSC for both the radiologist (CT: 0.81±0.15, T1-weighted MRI: 0.79±0.19) and medical student (CT: 0.84±0.14, T1-weighted MRI: 0.84±0.15). After excluding these samples, significant differences remained only for T1-weighted MRI for the radiologist (T1-weighted MRI: p=0.006). Qualitative scoring from the radiologist showed that most segmentations were deemed Sufficient or Excellent (CT: n=30/39, T1-weighted MRI: n=42/63), see **Table S5**.

The performances with real interactions by radiologists were investigated across different patient demographics including age (<18 vs. ≥18), tumor location (extremity vs. non-extremity) and disease types. For this analysis, cases with wrongly identified objects were excluded. No substantial differences in performance were observed for age (<18 age: 0.70±0.29, ≥18 age: 0.75±0.25) and tumor location (extremity: 0.79±0.20, non-extremity: 0.72±0.28). There were differences between disease types (GIST: 0.79±0.17, Schwannoma: 0.90±0.04, Leiomyoma: 0.90±0.03, Leiomyosarcoma: 0.83±0.12, Lipoma: 0.81±0.11, MLS: 0.69±0.28, DTF: 0.67±0.30, Myxofibrosarcoma: 0.81±0.07, WDLS: 0.88±0.06). These findings indicate potential reduced performance in specific disease types, such as desmoid-type fibromatosis (DTF). It is important to note that the sample sizes for this analysis were small, so conclusions should be drawn carefully.

**Supplementary Material C: Discussion**

**Applications in STT for time-efficient segmentation**

The ability to provide time-efficient segmentation on CT and MRI is important for the translation of morphological, volumetric quantifications, and radiomics biomarkers to clinical practice.

For example, segmentations are required for targeted (neo)adjuvant radiotherapy (3). Currently, these have to be made manually for each lesion at each regiment, which is a substantial burden on the physician’s time and drives healthcare costs. Also, the use of quantitative imaging features (i.e., radiomics) has often been described to provide accurate, noninvasive imaging biomarkers (4). In STT, radiomics has been used to predict phenotype (5-7) grading (22-24), and patient outcome (24-26). However, a major hurdle for translation of radiomics to clinical practice is the requirement for manual segmentation, which is observer dependent, time-consuming and therefore not feasible in the radiologist’s workflow. Recent work by Crombé et al., 2020, and Gitto et al., 2021, showed that none of the 52 and 49 included radiomics studies, respectively, were performed with automatic segmentation (27, 28). Only 13.5% and 8.0% used semi-automatic methods, and unlike the minimally interactive approach provided here, these methods still required intensive manual correction by the clinician. Finally, routine clinical measurements, such as the Response Evaluation Criteria In Solid Tumors (RECIST), would benefit from segmentation to reduce observer variability, or allow extension to 3D volume measurements, which has been shown to improve monitoring of therapy response (8, 29). Together, these examples highlight the need for (semi-)automatic segmentation in clinical practice.

Further research should explore the use of minimally interactive segmentation for these applications including the impact on the clinical workflow and decision making.

**Considerations regarding clinical implementation**

Here, we delve into the practical considerations for implementing the interactive segmentation method in clinical practice. Specifically, we address real user interactions, the implications of quality scoring, and the role of fully automatic segmentation.

In this study, synthetic interactions were derived from the reference segmentations to train and validate the method, since using real interactions would have been time-consuming due to the large number of patients. To verify that synthetic interactions reflected those of humans, both types were assessed on the WORC test dataset. Differences were found between real and synthetic interactions for the radiologist and medical student. However, this was mostly explained by segmentation of the wrong object by the user, as removing these samples yielded better results for both users. Furthermore, it is worth noting that in real clinical workflows, clinicians have access to additional information and multiple MRI sequences, reducing the likelihood of tumor misidentification compared to the setting used in our study with only the T1-weighted MRI data. Furthermore, the comparison with a medical student demonstrated that even users with limited knowledge of STT imaging characteristics can effectively utilize the method. However, it remains crucial to evaluate the method with a more diverse group of users who are representative of clinical workflow, such as general and abdominal radiologists. Additionally, further research should explore the performance of the interactive segmentation method within real clinical settings to assess its practical impact.

Moreover, users were restricted to six interactions. Allowing additional interactions would increase the interaction time, but could potentially enhance the exponentialized geodesic distance (EGD) map, thereby improving the differentiation between tumor voxels and surrounding tissue. This could be particularly beneficial for complex, multi-lobulated, and irregular tumors, which are currently the most challenging cases to segment. Future research could evaluate whether the extra time needed for additional interactions is worth the increase in accuracy.

Next, aside from comparing interactive segmentations to reference segmentations using DSC, interactive segmentation based on real interactions were also quality scored by the user. Both users scored most segmentations as Excellent or Sufficient. Future research should investigate the implications of these quality scores for clinical measurements; e.g. targeted radiotherapy might require Excellent segmentation while radiomics might perform adequately on Sufficient segmentations. Furthermore, still a substantial number of segmentations were scored as Insufficient, suggesting that manual correction may still be necessary in these cases. Nevertheless, interactive segmentation, where radiologists are already involved, may align more naturally with clinical workflows compared to fully automatic segmentation methods when adjustments are required.

Finally, even though our primary focus was on developing and implementing interactive segmentation, we also developed a fully automatic segmentation method for STT. While interactive segmentation generally outperformed fully automatic segmentation, the fully automatic method can still be valuable for certain patients, such as those with lipomas or leiomyosarcomas. Nevertheless, with fully automatic segmentation, the user should be wary for segmentation of the wrong object (CT: n=10/39, T1-weighted MRI: n=3/62). Therefore, expert knowledge is required regardless of the segmentation method applied.

**Reference**

21. Diaz-Pinto A, Alle S, Ihsani A, Asad M, Nath V, Pérez-Garcia F, et al. MONAI Label: A framework for AI-assisted Interactive Labeling of 3D Medical Images. arXiv preprint arXiv:2203.12362. 2022 Mar 23.

22. Peeken JC, Spraker MB, Knebel C, Dapper H, Pfeiffer D, Devecka M, et al. Tumor grading of soft tissue sarcomas using MRI-based radiomics. EBioMedicine. 2019;48:332–40.

23. Zhang Y, Zhu Y, Shi X, Tao J, Cui J, Dai Y, et al. Soft tissue sarcomas: preoperative predictive histopathological grading based on radiomics of MRI. Acad Radiol. 2019;26(9):1262–8.

24. Peeken JC, Bernhofer M, Spraker MB, Pfeiffer D, Devecka M, Thamer A, et al. CT-based radiomic features predict tumor grading and have prognostic value in patients with soft tissue sarcomas treated with neoadjuvant radiation therapy. Radiother Oncol. 2019;135:187–96.

25. Spraker MB, Wootton LS, Hippe DS, Ball KC, Peeken JC, Macomber MW, et al. MRI radiomic features are independently associated with overall survival in soft tissue sarcoma. Adv Radiat Oncol. 2019;4(2):413–21.

26. Crombé A, Le Loarer F, Sitbon M, Italiano A, Stoeckle E, Buy X, et al. Can radiomics improve the prediction of metastatic relapse of myxoid/round cell liposarcomas? Eur Radiol. 2020;30(5):2413–24.

27. Crombé A, Fadli D, Italiano A, Saut O, Buy X, Kind M. Systematic review of sarcomas radiomics studies: Bridging the gap between concepts and clinical applications? Eur J Radiol. 2020 Nov;132:109283.

28. Gitto S, Cuocolo R, Albano D, Morelli F, Pescatori LC, Messina C, et al. CT and MRI radiomics of bone and soft-tissue sarcomas: a systematic review of reproducibility and validation strategies. Insights Imaging. 2021 Jun;12(1).

29. Mozley PD, Bendtsen C, Zhao B, et al. Measurement of tumor volumes improves RECIST-based response assessments in advanced lung cancer. Transl Oncol. 2012;5(1):19-25.

**Supplementary Tables**

**Supplementary Table S1: Properties of the acquisition protocols**

| Protocol | WORC training dataset  (n = 412) [15] | | WORC test dataset (n = 102) [15] | | TCIA test dataset  (n = 51) [16] | |
| --- | --- | --- | --- | --- | --- | --- |
| Sequence | T1-weighted MRI (n=254) | CT (n=158) | T1-weighted MRI (n=63) | CT (n=39) | T1- and T2-weighted FS MRI (n=51) | CT (n=51) |
| Manufacturer |  |  |  |  |  |  |
| Siemens | 110 | 71 | 27 | 19 | 8 |  |
| Philips | 102 | 48 | 25 | 10 | 8 |  |
| General Electric | 42 | 7 | 11 |  | 33 | 51 |
| Canon |  | 29 |  | 7 |  |  |
| Toshiba |  | 3 |  | 3 |  |  |
| Varian |  |  |  |  | 2 |  |
| Magnetic field strength |  |  |  |  |  |  |
| 1T | 22 | - | 8 | - | NR | - |
| 1.5T | 211 | - | 52 | - | NR | - |
| 3T | 21 | - | 3 | - | NR | - |
| Slice thickness (mm)* | 4.67 ± 1.27 | 4.21 ± 1.17 | 4.77 ± 1.61 | 4.20 ± 1.09 | 5.70 ± 1.32 | 3.80 ± 0.00 |
| Pixel spacing (mm)* | 0.67 ± 0.25 | 0.75 ± 0.09 | 0.72 ± 0.33 | 0.72 ± 0.07 | 0.77 ± 0.32 | 0.98 ± 0.00 |

Note. – Abbreviations: T: tesla, NR: Not reported. FS: fat-saturated.
* Data are mean ± standard deviation (SD) in seconds.

**Supplementary Table S2: Rule-based scoring of tumor segmentation based on visual inspection by the user**

| Segmentation score | Definition |
| --- | --- |
| Excellent | The segmentation is almost perfectly aligned with the tumor and requires no adjustments. For this score, the segmentation volume should overlap with the tumor for at least 95%. |
| Sufficient | The segmentation is aligned with the tumor, however, could benefit from minor adjustments. For this score, the segmentation volume should overlap with the tumor for at least 75%. |
| Insufficient | The segmentation misses parts of the tumor, or parts are overlapping with normal tissue, therefore major adjustments are required. For this score, the segmentation volume should overlap with the tumor for at least 50%. |
| Incorrect | The segmentation is not overlapping with the tumor, or missing large areas of the tumor. For this score, the segmentation volume overlaps with the tumor for less than 50%. |

Note. – If the tumor cannot be located in the image, the clinician can score as “Cannot locate tumor”.

**Supplementary Table S3: Phenotype-specific agreement between nnU-Net, MIDeepSeg, and InteractiveNet with reference segmentation.**

|  | Sensitivity | | | Intersection over union | | |
| --- | --- | --- | --- | --- | --- | --- |
|  | nnU-Net | MIDeepSeg | InteractiveNet | nnU-Net | MIDeepSeg | InteractiveNet |
| **WORC test dataset** |  |  |  |  |  |  |
| *CT* |  |  |  |  |  |  |
| GIST | 0.51±0.46 | 0.89±0.08 | 0.88±0.11 | 0.44±0.41 | 0.64±0.25 | 0.74±0.10 |
| Schwannoma | 0.75±0.43 | 0.93±0.05 | 0.90±0.14 | 0.68±0.38 | 0.64±0.34 | 0.78±0.15 |
| Leiomyoma | 0.24±0.34 | 0.90±0.11 | 0.86±0.11 | 0.22±0.32 | 0.51±0.28 | 0.74±0.18 |
| Leiomyosarcoma | 0.69±0.40 | 0.87±0.06 | 0.81±0.11 | 0.64±0.38 | 0.69±0.17 | 0.76±0.13 |
| *Total* | 0.53±0.45 | 0.89±0.08 | 0.87±0.11 | 0.47±0.40 | 0.63±0.25 | 0.75±0.16 |
| *T1-weighted MRI* |  |  |  |  |  |  |
| Lipoma | 0.76±0.38 | 0.86±0.04 | 0.89±0.06 | 0.67±0.35 | 0.73±0.13 | 0.83±0.08 |
| MLS | 0.80±0.32 | 0.77±0.18 | 0.85±0.21 | 0.65±0.37 | 0.64±0.16 | 0.74±0.18 |
| DTF | 0.54±0.41 | 0.62±0.12 | 0.78±0.15 | 0.42±0.36 | 0.50±0.12 | 0.63±0.20 |
| Myxofibrosarcoma | 0.75±0.35 | 0.65±0.14 | 0.89±0.09 | 0.60±0.31 | 0.54±0.22 | 0.71±0.15 |
| WDLS | 0.85±0.28 | 0.78±0.09 | 0.88±0.08 | 0.82±0.28 | 0.66±0.21 | 0.81±0.10 |
| Leiomyosarcoma | 0.86±0.10 | 0.67±0.08 | 0.83±0.08 | 0.79±0.09 | 0.61±0.08 | 0.78±0.07 |
| *Total* | 0.74±0.34 | 0.72±0.14 | 0.85±0.12 | 0.64±0.34 | 0.61±0.18 | 0.74±0.15 |
| **TCIA test dataset** |  |  |  |  |  |  |
| *CT* |  |  |  |  |  |  |
| Liposarcoma | 0.70±0.35 | 0.78±0.14 | 0.84±0.13 | 0.60±0.32 | 0.61±0.16 | 0.75±0.13 |
| Leiomyosarcoma | 0.74±0.34 | 0.73±0.18 | 0.84±0.11 | 0.62±0.32 | 0.59±0.17 | 0.78±0.09 |
| Fibrosarcoma^§^ | 0.60±0.37 | 0.48±0.13 | 0.65±0.20 | 0.57±0.36 | 0.45±0.12 | 0.62±0.18 |
| Synovial sarcoma | 0.64±0.40 | 0.78±0.14 | 0.83±0.10 | 0.51±0.39 | 0.61±0.14 | 0.78±0.09 |
| MFH | 0.62±0.34 | 0.77±0.13 | 0.82±0.13 | 0.52±0.29 | 0.56±0.17 | 0.71±0.13 |
| ESBS | 0.57±0.38 | 0.75±0.15 | 0.81±0.11 | 0.53±0.35 | 0.56±0.16 | 0.68±0.13 |
| Other | 0.67±0.30 | 0.69±0.19 | 0.83±0.08 | 0.55±0.34 | 0.63±0.13 | 0.78±0.07 |
| *Total* | 0.66±0.35 | 0.75±0.15 | 0.83±0.12 | 0.56±0.32 | 0.58±0.16 | 0.74±0.12 |
| *T1-weighted MRI* |  |  |  |  |  |  |
| Liposarcoma | 0.70±0.35 | 0.78±0.14 | 0.84±0.13 | 0.60±0.32 | 0.61±0.16 | 0.75±0.13 |
| Leiomyosarcoma | 0.74±0.34 | 0.73±0.18 | 0.84±0.11 | 0.62±0.32 | 0.59±0.17 | 0.78±0.09 |
| Fibrosarcoma^§^ | 0.60±0.37 | 0.48±0.13 | 0.65±0.20 | 0.57±0.36 | 0.45±0.12 | 0.62±0.18 |
| Synovial sarcoma | 0.64±0.40 | 0.78±0.14 | 0.83±0.10 | 0.51±0.39 | 0.61±0.14 | 0.78±0.09 |
| MFH | 0.62±0.34 | 0.77±0.13 | 0.82±0.13 | 0.52±0.29 | 0.56±0.17 | 0.71±0.13 |
| ESBS | 0.57±0.38 | 0.75±0.15 | 0.81±0.11 | 0.53±0.35 | 0.56±0.16 | 0.68±0.13 |
| Other | 0.67±0.30 | 0.69±0.19 | 0.83±0.08 | 0.55±0.34 | 0.63±0.13 | 0.78±0.07 |
| *Total* | 0.66±0.35 | 0.75±0.15 | 0.83±0.12 | 0.56±0.32 | 0.58±0.16 | 0.74±0.12 |
| *T2-weighted FS MRI* |  |  |  |  |  |  |
| Liposarcoma | 0.70±0.35 | 0.78±0.14 | 0.84±0.13 | 0.60±0.32 | 0.61±0.16 | 0.75±0.13 |
| Leiomyosarcoma | 0.74±0.34 | 0.73±0.18 | 0.84±0.11 | 0.62±0.32 | 0.59±0.17 | 0.78±0.09 |
| Fibrosarcoma^§^ | 0.60±0.37 | 0.48±0.13 | 0.65±0.20 | 0.57±0.36 | 0.45±0.12 | 0.62±0.18 |
| Synovial sarcoma | 0.64±0.40 | 0.78±0.14 | 0.83±0.10 | 0.51±0.39 | 0.61±0.14 | 0.78±0.09 |
| MFH | 0.62±0.34 | 0.77±0.13 | 0.82±0.13 | 0.52±0.29 | 0.56±0.17 | 0.71±0.13 |
| ESBS | 0.57±0.38 | 0.75±0.15 | 0.81±0.11 | 0.53±0.35 | 0.56±0.16 | 0.68±0.13 |
| Other | 0.67±0.30 | 0.69±0.19 | 0.83±0.08 | 0.55±0.34 | 0.63±0.13 | 0.78±0.07 |
| *Total* | 0.66±0.35 | 0.75±0.15 | 0.83±0.12 | 0.56±0.32 | 0.58±0.16 | 0.74±0.12 |

Data are mean ± standard deviation (SD). FS: fat-saturated; MLS: myxoid-liposarcoma; DTF: desmoid-type fibromatosis; WDLS: well-differentiated liposarcoma; GIST: gastrointestinal stromal tumor; MFH: malignant fibrous histiocytoma; and ESBS: Extraskeletal bone sarcoma.

^§^ Standard deviation could not be calculated as only one fibrosarcoma was present in the TCIA test dataset.

**Supplementary Table S4: Comparison of interactive segmentation performance using interactions by different annotators.**

|  | Radiologist | Medical student | Synthetic | P value for synthetic vs radiologist* | P value for synthetic vs student* | P value for radiologist vs student* |
| --- | --- | --- | --- | --- | --- | --- |
| CT |  |  |  |  |  |  |
| All tumors | 0.77 ± 0.24 | 0.60 ± 0.40 | 0.85 ± 0.11 | .36 | .02 | .03 |
| Correctly identified tumors† | 0.81 ± 0.15 | 0.84 ± 0.14 |  | .09 | .29 | .99 |
| T1-weighted MRI |  |  |  |  |  |  |
| All tumors | 0.75 ± 0.25 | 0.77 ± 0.27 | 0.84 ± 0.12 | .007 | .27 | .002 |
| Correctly identified tumors† | 0.79 ± 0.19 | 0.84 ± 0.15 |  | .006 | .55 | <.001 |

Note. – Except where indicated, data are mean ± standard deviation (SD) for the Dice Similarity Coefficient (DSC). Results are reported on the WORC test dataset.

***** P-values are reported for the Wilcoxon signed-rank test.

† Number of correctly identified tumors differ between radiologist and student.

**Supplementary Table S5: Quality scoring determined through visual inspection of the interactive segmentation made using interactions by different annotators.**

|  | CT | | T1-weighted MRI | |
| --- | --- | --- | --- | --- |
|  | Radiologist | Medical Student | Radiologist | Medical Student |
| Excellent | 18 | 4 | 16 | 10 |
| Sufficient | 12 | 22 | 26 | 30 |
| Insufficient | 7 | 7 | 16 | 15 |
| Incorrect | 2 | 3 | 3 | 4 |
| Cannot locate tumor | 0 | 3 | 1 | 3 |

Note. – Results are reported on the WORC test dataset.

**Supplementary Figures**


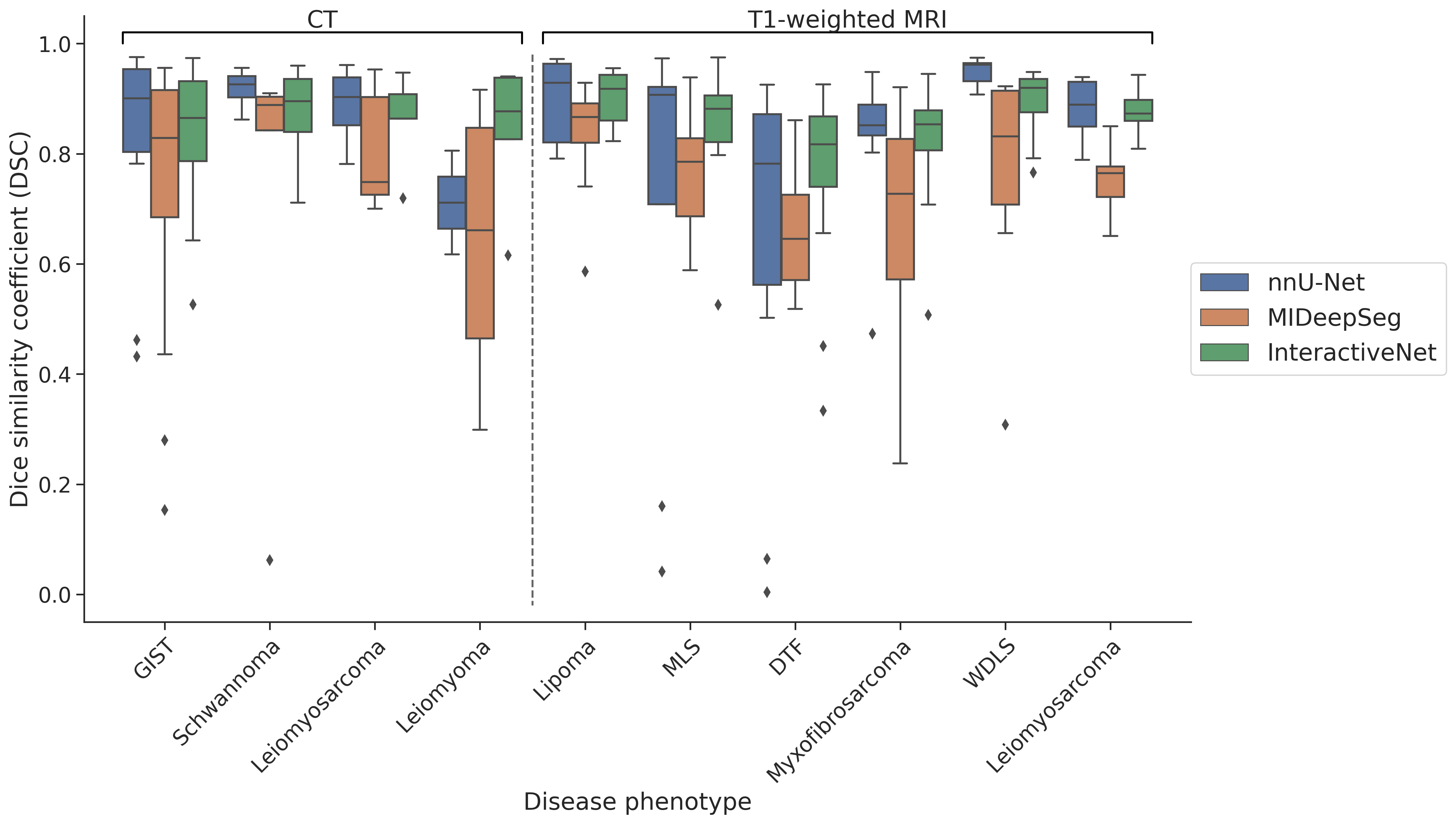


Supplementary Figure S1: Quantitative results from automatic and interactive segmentation of STTs on the WORC test dataset after removing the 23 outliers on which the automatic method failed to segment the correct lesion. Box-and-whisker plot, visualizing the median, quartiles, and potential outliers, for the Dice Similarity Coefficient (DSC) results of fully automatic nnU-Net (blue), interactive ‘MIDeepSeg’ (orange), and proposed ‘InteractiveNet’ (green) segmentation methods for different phenotypes on CT and MRI. DTF: desmoid-type fibromatosis; WDLS: well-differentiated liposarcoma; and GIST: gastrointestinal stromal tumor.


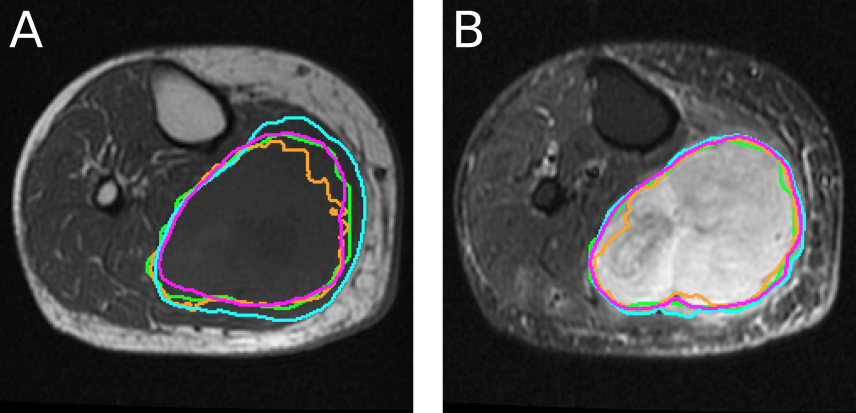


Supplementary Figure S2: Example from the TCIA test dataset with segmentation methods on A) T1-weighted MRI in the transverse plane, B) T2-weighted fat-saturated MRI in the transverse plane. The reference segmentation is displayed in green, InteractiveNet (proposed interactive) segmentation in pink, MIDeepSeg (interactive) segmentation in orange, and nnU-Net (fully automatic) segmentation in cyan. The contrast in the T2-weighted fat-saturated image between the tumor and surrounding tissue improved both segmentation results.





Supplementary Figure S3: Scatterplots for volume and diameter measurements from automatic and interactive segmentation methods compared to reference segmentations of STTs on A) the WORC test dataset, and B) the TCIA test dataset. Every dot represents a patient. Patients are color-coded based on their achieved Dice Similarity Coefficient (DCS) using the respective method, i.e. automatic or interactive segmentation. The dotted line represents a perfect agreement.


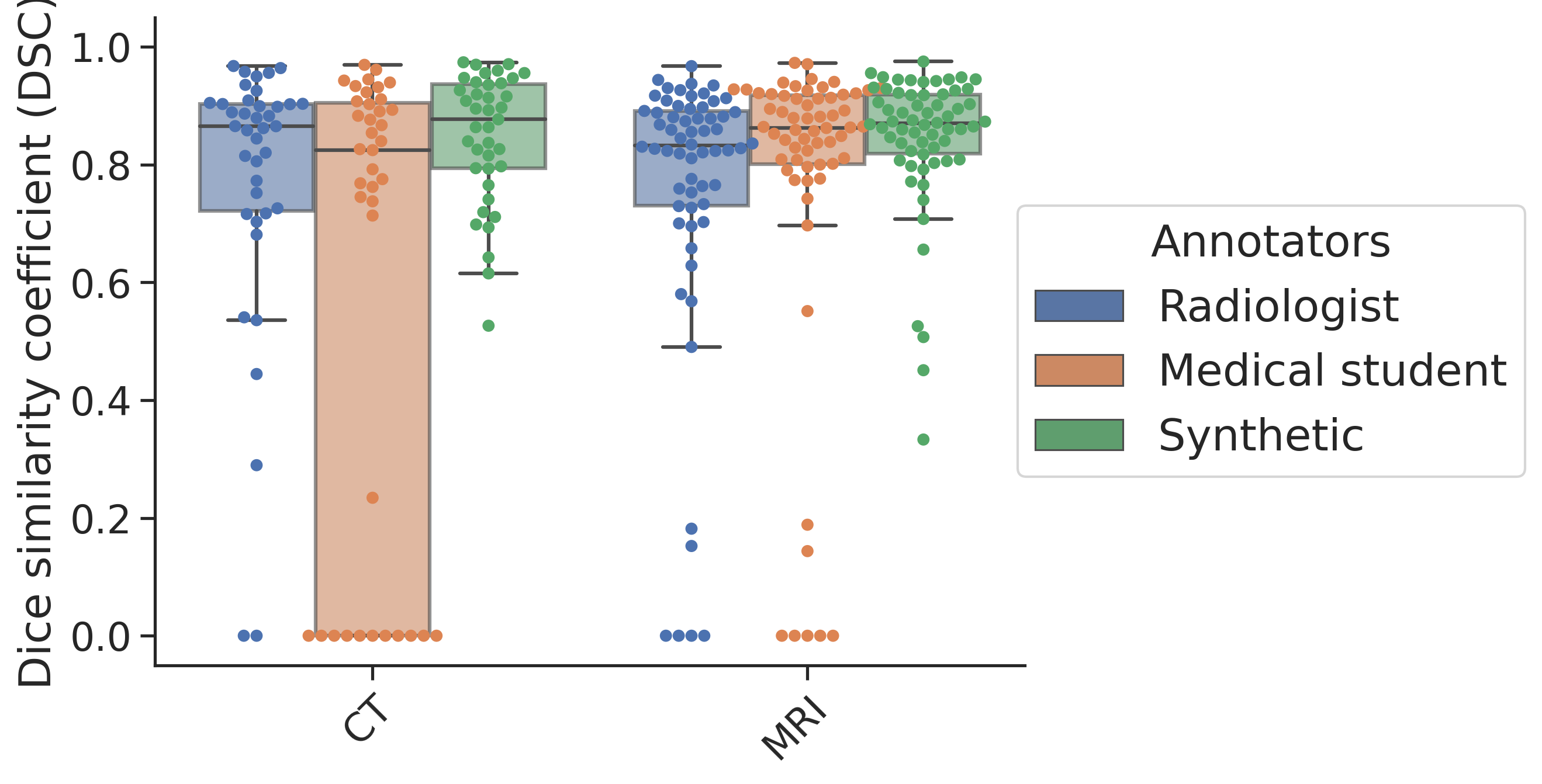


Supplementary Figure S4: Quantitative comparison of predicted segmentations compared to reference segmentations using the interactive segmentation method for different annotators. The annotator is either the musculoskeletal radiologist (blue), medical student (orange), or synthetic annotation (green). Every dot represents a sample in the WORC test dataset.

**Supplementary Video**

**Supplementary Video S1:** Tutorial of the InteractiveNet workflow in 3D slicer. Example shown in the demonstration is an atypical lipomatous tumor on T1-weighted MRI.
